# Migrants’ Social Integration and Its Relevance for National Identification: An Empirical Comparison Across Three Social Spheres

**DOI:** 10.3389/fsoc.2021.700580

**Published:** 2022-01-03

**Authors:** Charlotte Clara Becker

**Affiliations:** Institute of Sociology and Social Psychology, University of Cologne, Cologne, Germany

**Keywords:** national identification, emotional integration, social integration, contact, Germany

## Abstract

A key element of migrants’ well-being is their emotional integration, that is, the extent to which they perceive themselves as members of society and their identification with the country they are living in. To foster this sense of belonging, many integration programs aim to increase the migrants’ social integration, for example, by organizing events for migrants to meet natives in various settings. The validity of this strategy is supported by decades of international research. It remains unclear, however, which aspects of social integration are most relevant for national identification. Multiple theories concerned with contact and group identification support the assumption that contact to natives should foster a sense of belonging and national identification. However, for a contact situation to bear this potential, a certain set of criteria, including aspects like direct personal contact, a similar social status, and the presence of egalitarian norms, needs to be fulfilled. It is expected that these characteristics are more likely to be fulfilled within family and friendship settings than in contact situations within the employment context. Hence, I expect contact to natives within the network of friends and family to be more greatly associated with migrants’ national identification. I analyzed data from a 2013 cooperation between the Institute for Employment Research (IAB) and the German Socio-Economic Panel (SOEP), that is, the IAB-SOEP Migration Sample, as well as the 2014 wave of the SOEP. The subsample used included 2,780 first- and second-generation migrants living in Germany. The results indicate that not all kinds of contact are equally linked to national identification. In contrast to expectations, in neither the cross-sectional models nor the lagged models was living together with native family members significantly linked to national identification. Similarly, the association between having predominantly native co-workers and national identification was insignificant when controlling for migrant-specific characteristics. Only the relation with having predominantly native friends was significant and positive across all models. This as well as a comparison of the associations lead to the conclusion that when it comes to migrants’ national identification native friends might be the most relevant form of contact to natives.

## Introduction

In recent decades the integration of migrants has become a prominent challenge for many Western and non-Western societies. And with this development more and more sociological research has focused on migrants’ integration as well. Many researchers in the past have focused on cultural, social, and structural integration, by asking which determinants lead to the assimilation of migrants’ concerning their knowledge and skills, their social networks, and their positions in society (e.g., Bevelander and Veenman, 2006; Martinovic et al., 2009a; Hochman, 2011; Fokkema and Haas, 2015; Kalmijn and Kraaykamp, 2018). Besides that, there is a line of research focusing on migrants’ emotional integration, namely their sense of belonging and identification with the new society they are a part of. However, within this line of research there are still many uncertainties. This is specifically true with regard to explaining migrants’ emotional integration with quantitative research using adult samples.

This is unfortunate because emotional integration is a key element for migrants’ well-being. In addition, emotional integration is not only of great relevance for the individual migrant; rather, the whole society benefits from migrants having high levels of emotional integration. This is because high levels of emotional integration, specifically national identification, can be considered the basis for national solidarity and an overall effective democracy (Barry, 2002; Putnam, 2007; Verkuyten and Martinovic, 2012). Existing studies on emotional integration have looked for key determinants. Social integration, that is, the contact to society members without a migration background, appears to be one of them. Within this area of contact to natives there have been, among others, studies on the contact to native neighbors (e.g. de Vroome et al., 2014), specific school settings (e.g. Agirdag et al., 2011), and the composition of friendship networks (e.g. Walters et al., 2007).

However, previous research focused on individual aspects of social integration rather than analyzing the influence of different aspects simultaneously and comparing their relevance for national identification. It is therefore unclear, which aspects of social integration are most relevant. Yet, to increase migrants’ national identification and, therefore, their well-being and to develop integration policies and programs, it is vital to understand which aspects of social integration are most relevant. Only when this knowledge is acquired can effective integration policies, programs, and interventions based on contact to natives be designed. My aim it to fill this gap by inspecting the influence of various kinds of contacts to natives on national identification simultaneously and comparing the respective effects empirically. Specifically, I will describe and compare the relationships between family, friendship, and workplace settings and national identification.

In a first step, I will present some theoretical considerations on the concept of emotional integration. Further, I will discuss previous studies on the relation of social and emotional integration. Based on this theoretical background and additional theories, concrete expectations for the results will be stipulated in form of testable hypotheses. Subsequently, after presenting the dataset used as well as the methods applied, I will analyze and compare the relationship between different forms of social integration and national identification. The article will conclude with a discussion of the results as well as potential limitations of the study.

### Emotional Integration and Integration Theory

Emotional integration has been described as the emotional relationship between a migrant and the social system (Esser, 2001). This emotional relationship can also be understood as the degree to which migrants hold a collective sense of togetherness or national pride (Esser, 2001; Hochman, 2010). Overall, emotional integration aims to capture migrants’ sense of belonging to the society of the country they are residing in.

In the last decades, different approaches to measure emotional integration have been developed. These include the widely accepted Collective Self-Esteem Scale (CSES) (Luhtanen and Crocker, 1992), which is based on Tajfel and Turner’s (1986) concept of social identity (see also Crocker et al., 1994; Kim and Omizo, 2005; Gangadharbatla, 2008; Agirdag et al., 2011). Another, less theory-driven, approach investigated migrants’ behavior to gather information on their emotional integration (Becker, 2009). Both approaches, however, can be difficult to implement because they require very specific data structures, which almost exclusively are obtained by data gathered for specific research projects. Another common and, in many cases, more feasible way to measure emotional integration is by means of national identification (Walters et al., 2007; Hochman, 2010; Maliepaard et al., 2010; Hochman and Davidov, 2014). Variables indicating respondent’s levels of national identification can often be found in large surveys, such as the European Social Survey (European Social Survey, 2018) or the German Socio-Economic Panel (SOEP), which asks respondents “To what extent do you feel German?” (TNS Infratest Sozialforschung, 2014). Throughout the rest of the article, national identification will be used to describe emotional integration unless otherwise specified.

Integration theories suggest a strong relation between other forms of integration and national identification (Esser, 2001; Nauck, 2001). These forms include: 1) structural integration—the migrants position in society and its core institutions (Heckmann, 2005), 2) cultural integration—the acquisition of knowledge, skills, attitudes and behaviors specific to a certain country or region (Heckmann, 2003) and 3) social integration—(regular) contact to and interactions with natives (Haug, 2003; Martinovic et al., 2009b). These relations are suggested to be causal, with national identification being seen at the end of the overall integration process (Esser, 2001; Nauck, 2001). This is because it is assumed that structural and cultural assimilation as well as general contact to natives increase migrants’ possibilities for participating in society and therefore help increase their sense of belonging and national identification (Esser, 2001).

### Previous Research on the Link Between Social Integration and National Identification

In the recent past, many studies investigating these relations supported the assumption of a close relationship between different forms of integration and national identification. Concerning structural integration, factors like naturalization, and entitlement to vote were identified as being relevant for migrants’ national identification (Ono, 2002; Walters et al., 2007; Fick, 2016), and with regard to cultural integration, language acquisition was found to be highly influential (Jasinskaja-Lahti and Liebkind, 1998; Remennick, 2004; de Vroome et al., 2014; Hochman and Davidov, 2014).

However, only few researchers were specifically interested in the effect of contact to natives on national identification in adults. Many researchers focused on children or when using adult samples merely included aspects of social integration as control variables into their models when analyzing the influence of other characteristics on national identification. Nonetheless, their work can be used to study the effect. Therefore, in the following, both studies explicitly focusing on the relation between social integration and national identification as well as studies indirectly contributing will be discussed.

Research including variables indicating the occurrence of contact to natives generally were able to show that migrants’ national identification was strongly linked to increased contact to natives in everyday life (Hochman, 2010; de Vroome et al., 2011; Tolsma et al., 2012; Hochman and Davidov, 2014). While these studies provide a first indication of the relevance of social integration, more information on the effect of contact in various settings is necessary to learn about the potential differences in the effects.

One setting that has been explored further with respect to the relation between social integration and national identification is the neighborhood. For instance, in a sample of Caribbean and South Asian migrants in Britain, Maxwell (2009) indicated that those living in ethnically diverse neighborhoods in two out of three measurement time points (2003, 2005) exhibited the same level of national identification as those living in neighborhoods comprised of only members of their own ethnicity. In 2007, those living in diverse neighborhoods were less likely to report high levels of national identification. However, due to the strict definition of diversity, this variable had little variance. In both ethnic groups as well as over the three time points, more than 91% of respondents reported living in diverse neighborhoods. Instead of looking at the general composition on the neighborhood, de Vroome and colleagues (2014) analyzed the relation between national identification and contact to native neighbors specifically. Whereas migrants indicating more contact to native neighbors showed significantly higher levels of national identification, this relation was not very strong and could only be observed in first-generation migrants (de Vroome et al., 2014). Second-generation migrants’ identification was not associated with the amount of contact to native neighbors, which was generally reported to be quite high across members of this group (de Vroome et al., 2014).

A more personal sphere, and the sphere most frequently applied, are migrants’ friends and friendship networks. Specifically, many studies utilized information on the ethnic composition of the migrants’ friendship networks (Lubbers et al., 2007; Walters et al., 2007; Maxwell, 2009; Hochman, 2010; Hochman and Davidov, 2014). In a sample of Puerto Ricans living in New York who reported the majority of their friends to be non-Hispanics, Oropesa et al. (2008) reported an increase in pan-ethnic over ethnic self-labeling. Similarly, Lubbers et al. (2007) found that with increasing percentages of native friends, the likelihood for immigrants in Spain to report generic rather than ethnic identification increased substantially. While these two studies focused on ethnic and pan-ethnic rather than national identification in the sense of identifying with the migrants’ (new) place of residency, Maxwell (2009) explicitly investigated the relation between migrants having friends outside their own ethnic group and their sense of belonging to Britain. As expected, migrants with interethnic friendship networks reported a considerably stronger sense of belonging to Britain than those whose friendship networks were exclusively intraethnic (Maxwell, 2009). Comparable results were also found for Canada and Germany, with respondents with higher shares of native friends also reporting higher levels of national identification (Walters et al., 2007; Hochman, 2010; Hochman and Davidov, 2014).

In addition to these studies on adult migrants, there is also a line of research focusing on national identification in migrant children and adolescents. While some studies in this field confirm the results found in studies with adults (Phinney et al., 2006; Sabatier, 2008; Agirdag et al., 2011; Munniksma et al., 2015; Fleischmann and Phalet, 2018), others did not find this relation in their samples (Leszczensky, 2013; Leszczensky et al., 2016; Leszczensky, 2018). Therefore, in children the relation between having native friends and national identification appears to be more disputed, especially since the studies indicating no relation tended to use longitudinal analyses strategies, which are more suitable for analyzing causation^1^. Another interesting result from studies focusing on children is that the significance and strength of the relation between friendship network composition and national identification might differ between ethnic groups (Leszczensky, 2013; Schulz and Leszczensky, 2016). While for young migrants with southern European and former Yugoslavian roots the relations were in line with those commonly found in adults, the effect was not statistically significant for those of Polish and Turkish descent (Schulz and Leszczensky, 2016). This indicates that different migration backgrounds should be accounted for when researching the link between having native friends and national identification.

Further, researchers considered the contact to natives within the migrants’ immediate families. This sphere is most likely the most personal one, as it considers migrants’ choice in relationship partners. Results from Germany as well as the Netherlands indicated that migrants having a native partner display substantially higher levels of national identification than those who have partners sharing the same ethnicity (Rother, 2008; de Vroome et al., 2011). With similar results, but a different approach focusing on emotional integration rather than national identification, Becker (2009) and Gerhards and Hans (2009) also came to the conclusion that those with native partners were more likely to be emotionally integrated. Besides having a native partner, living together with a native in general appeared to have a positive effect on national identification (Fick, 2016).

As can be deduced from the presented studies, most research focused on spheres in which migrants are able to choose whether to have contact to specific others or not to engage in such contacts. Having a native partner and having native friends are choices made by the individual. Similarly, having contact to natives within the neighborhood can also be seen as a voluntary act, however, perhaps to a lesser extent than that of friendship and partner formation. To compare the relevance of different spheres, it would also be interesting to discuss settings in which migrants have no or relatively little influence on the composition of their network. One example for such a setting would be their place of work and the ethnic composition of their colleagues. Unfortunately, this context has been neglected to date. What exists, however, is research on the relation between schools’ ethnic compositions and the national identification of adolescent migrants (Sabatier, 2008; Agirdag et al., 2011). Similar to situations where employees have little influence on the ethnic composition of the workforce they are a part of, pupils have little influence on the school and class composition. Therefore, results from studies conducted in schools could give first insights into the relevance of contact to natives in settings in which no or little individual choice concerning the contact is possible. Two studies conducted in Belgium and France used the percentage of native students at school as explanatory variables (Sabatier, 2008; Agirdag et al., 2011). Both came to the conclusion that while the composition initially appeared to be relevant for the national identification of students with a migration background, after controlling for aspects such as interethnic friendships and ethnicity, the relation lost its statistical significance (Sabatier, 2008; Agirdag et al., 2011). This would suggest that when controlling for other factors, the ethnic composition of networks in which individuals do not choose their counterparts might exhibit no or only a weak effect when compared to contact in other settings.

However, while the composition of the school did not exert statistically significant correlations with national identification, both studies highlight the relevance of friendships formed between migrant and native adolescents (Sabatier, 2008; Agirdag et al., 2011). This emphasizes the importance of including multiple aspects of social integration into analyses. Besides Sabatier (2008) and Agirdag et al. (2011) other researchers also opted for the inclusion of multiple aspects. De Vroome et al. (2011), for example, included three different aspects into their analyses on refugees national identification—whether they had at least one Dutch friend, whether half or more of their general social contacts were Dutch, and whether they had a Dutch partner. All three aspects correlated with national identification to a noteworthy degree and on a statistically significant level. Similarly, Gerhards and Hans (2009) included general contact (being visited by or having received visits from native Germans) as well as friendship aspects (at least one of the closest three friends is native German) and inter-marriage into their models. Again, all three aspects were substantial and statistically significant; however, intermarriage lost its statistical significance upon controlling for German citizenship. In neither of the two studies were simple comparisons of the coefficients possible, as no standardized effect sizes were provided and the authors did not discuss the differences in the coefficients size. Hochman and Davidov (2014), however, did include standardized effects in their work on the relation of language proficiency and national identification. As control variables they included the general visitation and friendship measures already used by Gerhards and Hans (2009). While both effects were substantially and statistically significant, the effect for general contact appeared to be slightly larger. However, it was not possible to determine whether this difference was statistically significant.

Overall, previous research has shown the importance of contact for migrants’ national identification across several different settings. From research including multiple factors, first indications for potential differences in the effects of various spheres can be drawn. However, due to the variations in the scale of the included variables, these comparisons suffer from limited comparability. In the next paragraphs I will discuss these differences from a theoretical perspective and frame concrete hypotheses.

### Three Theories of Social Integration

First assumptions on the relation between social integration and national identification can be drawn from the concept of social distance. Social distance is seen as a subjective measure describing the perceived void toward another person or social group (Ouellette-Kuntz et al., 2010). One example for this distance could be the affiliation to different social classes or, as relevant here, to being a member of the perceived category migrant versus the category native. Within groups, defined by sharing the same differentiator, there is little social distance between the individuals and the members feel a sense of belonging (Hill, 1984). It can be expected that migrants who have social networks similar to that of natives, that is, networks including large shares of natives, perceive a smaller social distance toward natives and the host society and, therefore, exhibit a greater sense of belonging.

The self-categorization theory (Turner et al., 1987), an advancement of the social identity theory (Tajfel and Turner, 1986), supports these assumptions. The theory assumes that individuals assign themselves to groups with whom they perceive to have similarities while at the same time they try to distance and demarcate themselves from groups with whom they perceive to have less in common (Turner et al., 1987). The knowledge of being a member of a group sharing certain characteristics not only increases the individual’s sense of belonging to said group (social distance) but also the individual’s identification with it (Turner et al., 1987). Applying this to the cases of migrants and their national identification, this means that increased similarity between a migrant and the native population should lead to an increased awareness of shared group membership and, hence, increased levels of national identification. Specifically, regarding the effect of social integration: With increased similarity of a migrant’s social network to social networks observed in natives (everyday contact to natives in various settings), the migrant’s national identification is expected to increase as well.

The contact theory further endorses these expectations, specifically with respect to the effect of social integration. The main idea of the contact theory is that interactions between groups or between individuals from different groups are necessary to dissolve group barriers that exist between them (Allport, 1954). The contact helps individuals to see each other as individuals rather than simple members of another, in itself uniform, group (Brewer and Miller, 1984). This process of individualization further leads to more positive attitudes toward the individuals as well as the individuals’ groups (Brewer and Miller, 1984). Concerning migrants’ national identification, this would indicate that migrants who have contact to natives develop a more positive attitude toward the host society. This might then influence their national identification since, with respect to the social identity theory, a positive attitude is considered a prerequisite for identification with a specific group (Tajfel and Turner, 1986). Considering all presented theories, a positive association between the amount of contact migrants have with natives and their levels of national identification can be expected (H1).

While all three theories indicate that increased contact to natives should lead to a higher sense of belonging as well as a higher level of national identification, further aspects need to be considered in order to evaluate the potential effects of contact in various settings. This is due to the fact that contact alone is not considered to be sufficient to provoke a lasting attitude change (Amir, 1969). To achieve the desired change in attitude and herewith identification, the contact needs to occur under specific conditions (Amir, 1969; Brewer and Miller, 1984). Advantageous characteristics for the contact setting are direct personal contact and the possibility to contest existing stereotypes, a similar social status of all individuals involved in the situation, the presence of egalitarian norms, and a collective goal that creates a cooperative interdependence between the individuals involved (Allport, 1954; Cook, 1978; Brewer and Miller, 1984). Further, the salience of the group membership in the contact situation affects the impact on the individuals attitudes (Hewstone et al., 1986; Brown et al., 1999; Voci and Hewstone, 2003; Kenworthy et al., 2005). Not all contact situations fulfill these requirements, but it can be assumed that contacts to natives that occur in settings which fulfill the requirements have a greater effect on migrants’ national identification than contacts occurring in settings in which the requirements are not fulfilled.

One contact setting that can be assumed to fulfill multiple of the mentioned characteristics is contact to natives within the family setting, for example, migrants who are married to a native partner. Within families, direct personal contact on a regular basis is given, and it can be assumed that the possibility to contest stereotypes is given as well. Family members are often named as individuals’ closest contacts and as the people with whom individuals discuss important issues (McPherson et al., 2006; Klofstad et al., 2009). Further, a similar social status between partners can be assumed because relationships tend to happen between individuals who are from similar social backgrounds (Kalmijn, 1998; McPherson et al., 2001; Blackwell and Lichter, 2004) and also because within-couple resources are commonly shared to a certain degree (Dew, 2008; Lyngstad et al., 2011). Interdependence is also given—on the one hand due to the shared resources and on the other hand because decisions made by individuals strongly influence the other members of the family (Kelley and Thibaut, 1978). While interdependence and intimate contact can hardly be disputed, the existence of egalitarian norms in contact settings within families is harder to describe. While gender norms and attitudes have changed toward egalitarianism across many Western societies, gender differences in housework and care work are only slowly reducing and, therefore, largely prevail until today (Hook, 2006; Scott, 2006; England, 2010; Altintas and Sullivan, 2017). Whether these differences are present across all contact situation within the family setting is questionable, however.

Similar to contact within the family, contact among friends usually fulfills the criterion of direct contact as well. Further, it probably provides a sphere which allows the contestation of stereotypes since research has shown that discussions among friends include very personal as well as intimate, but also political topics (Aries and Johnson, 1983; Diiorio et al., 1999). There are indications that political discussions taking place in settings that encourage small talk and provide room for general social interactions and bonding are more likely to change participants opinions and to foster an understanding for others (Kligler-Vilenchik, 2021). Additionally, a similar social status between friends is likely, given there is a general tendency toward homophily in friendships networks (Verbrugge, 1977; McPherson et al., 2001; Burgess et al., 2011). The similar status seems to be accompanied by egalitarian norms, friendships tend to be horizontally organized and based on equality (Laursen and Bukowski, 1997; Berenskoetter, 2014), with friendships exhibiting power differences being perceived as of lesser quality (Veniegas and Peplau, 1997). Another argument for egalitarian norms in friendship settings is the possibility for all involved to dissolve the friendship and build new ones if the relationship is perceived to be unequally beneficial. Lastly, a greater collective goal as well as interdependence are difficult to judge in the friendship context, and research on the issue is sparse. It can be assumed that interdependence is not as great as in the family setting, since friends’ decisions potentially have less influence on the lives of other friends than on (close) family members.

Concerning the workplace, a collective goal and a certain level of interdependence can be assumed. Co-workers work on projects together and the success of the project or company depends on the whole workforce and not just the individual worker. Besides that, employees might also depend on fellow co-workers to fulfill their duties and obligations so that one’s own responsibilities can be fulfilled. However, even though there is interdependence and contact at the workplace tends to be personal contact, it is questionable whether the possibility to challenge stereotypes exists. When asked about the individuals with whom one discusses matters that are important, people rarely named their co-workers (Klofstad et al., 2009). Likewise, the presence of a similar social status is debatable since workplace environments are often based on hierarchical structures and power asymmetries. This might also impact the existence of egalitarian norms. Their existence is further questioned with respect to migrants in the workforce given that workplace racism and discrimination are common phenomena (Deitch et al., 2003; Rospenda et al., 2009; Kahanec et al., 2012; Rosette et al., 2018).

Overall, it appears that most of the conditions would be fulfilled within the family setting. Similarly, contact to friends is likely to occur under conditions advantageous for positive attitude and identification change. The workplace environment, on the other hand, seems to fulfill only some of the conditions mentioned above. This leads to the following hypotheses:

The association between having native family members and national identification is stronger than the link between having native friends and national identification (H2).

The association between having native family members and national identification is stronger than the link between working together with natives and national identification (H3).

The association between having native friends and national identification is stronger than the link between working together with natives and national identification (H4).

## Materials and Methods

### Dataset

The analyses presented in this article use data from the IAB-SOEP Migration Sample collected in 2013 (Brücker et al., 2014). The dataset stems from a collaboration between the IAB (Institute for Employment Research) in Nuremberg and the SOEP at the German Institute for Economic Research in Berlin. It includes migrants who immigrated to Germany after 1994 as well as individuals with a migration background who were born in Germany as anchors. Additionally, interviews were conducted with the anchors’ household members who were over the age of 16. In total, the dataset includes 4,964 respondents, mostly first- and second-generation migrants. Since it was not possible to differentiate later migrant generations from respondents without a migration background, both groups had to be excluded from the analyses. Further, students, those who were completing an apprenticeship trainee or a voluntary social year and those who were unemployed (including most retirees) as well as part-time retirees working zero hours were excluded from the analyses. For most of these excluded individuals, no information on the contact to natives within the workplace environment was available^2^. Appendix A1 provides detailed information on the number of respondents excluded from the final sample for each of the above-described categories. The final sample included in the analyses is *N* = 2,780.

Respondents from the 2013 IAB-SOEP Migration Sample were invited to become members of the SOEP panel in the following year (Liebig et al., 2019). While later waves of the SOEP do not include large numbers of migration-specific variables, aspects of national identification are regularly included in the questionnaire. I merged the 2014 wave of the SOEP to the generated dataset, and 1,943 participants could be matched. Generally, many respondents from the IAB-SOEP Migration Sample chose not to participate in later waves of the SOEP.

### Variables

At both time points, the dependent variable *national identification* was measured by the item “To what degree do you think of yourself as German?” with response categories ranging from 1 (completely) to 5 (not at all). The variable was recoded, so that higher values indicate greater national identification. In the following sections, *national identification 2013* refers to the data collected with the IAB-SOEP Migration Sample in 2013 and *national identification 2014* refers to the information gathered from those individuals who also participated in the 2014 wave of the SOEP. Besides national identification, information for all other variables was taken from the IAB-SOEP Migration Sample (2013).

Contact to natives within the family setting was operationalized as having a *native family* member, that is, a household member who is not a first- or second-generation migrant. Respondents who had a native family member were assigned the value 1, and respondents who had no native household members or only household members with a migration background were assigned the value 0.

Contact to natives within the friendship setting was measured by the variable *mostly native friends.* Respondents were assigned the value 1 if about one-quarter, less than one-quarter, or none of their friends were foreigners, and they were coded 0 if about half, most, or all of their friends were foreigners.

A similar operationalization was used for contact to natives in the workplace setting (*mostly native work*). Respondents were assigned the value 1 if about one-quarter, less than one-quarter or none of the staff at their workplace were foreigners, and they were coded 0 if about half, most, or all of their fellow staff members were foreigners.^3^

As control variables I included various aspects, including sociodemographic as well as migration specific characteristics. Besides *age*, calculated by subtracting the respondents birth year from the time point of data collection, and gender (*male* = 1, female = 0), level of education was included into the analyses. Because no information on the years of schooling was available for most migrants, two dummy variables using the available ISCED-2011 (International Standard Classification of Education) coding scheme were created. *Secondary education* was designated as 1 for respondents whose highest degree came from an institution of secondary education and 0 for everyone else. *Higher education* was designated as 1 for respondents who reported having an educational qualification that exceeded the secondary level and 0 for everyone else. Participants who only received a primary education formed the reference category. In addition to aspects of education, I also considered aspects of the employment situation by including variables controlling for *part-time* (1 if part-time employee, 0 otherwise) and *marginal employment* (1 if marginally employed, 0 otherwise). The reference category was comprised of individuals working full-time.

Concerning the migrant-specific variables, I included *language skills* as an index of the reversed self-reported writing, reading, and speaking skills (each 0–5). The overall scale varied between 0 and 15, with higher values indicating a better understanding of the German language. In addition, I included *second-generation*, a variable indicating whether a respondent was a first- or second-generation migrant (1 if respondent was born in Germany, 0 if respondent was born abroad), *German citizenship* (1 if respondent had the German citizenship, 0 otherwise) as well as the migrants’ region of origin. The latter was operationalized as dummy variables following an allocation scheme by Seibert (2011), which first considers the respondents’ citizenship, prior citizenship, second citizenship (where applicable), and if necessary the parent’s citizenship. If this information did not support the regional allocation, the respondent’s and his or her parent’s birthplace were used. The following regions of origin were differentiated: *Turkey*, member states of the Commonwealth of Independent States (*CIS*), countries from the *Arab League*, and *Other origins*. Migrants from the European Union (EU) built the reference category. Details on the specific countries belonging to each category can be found in Supplementary Appendix S2.

### Methods

To test the hypotheses, I ran ordered logit regressions. As a starting point I used cross-sectional models only including information from the IAB-SOEP Migration Sample. Model 1.1 uses *national identification 2013* as the dependent variable and only includes the three contact variables as independent variables. Model 1.2 also includes the general control variables, and Model 1.3 includes all three contact variables as well as general and migrant-specific control variables. For better comparisons between the three models, all three used the same sample, meaning that only those respondents for whom information on all variables was available (those included in Model 1.3) contributed. Also, to increase comparability between the three contact variables, they were standardized in all models.

In a second step, I ran the same regressions now using *national identification 2014* as the dependent variable. All other variables (contact and control) stem from the IAB-SOEP Migration Sample from 2013. Again, I ran three models following the above-described scheme (Model 2: Model 2.1, Model 2.2, and Model 2.3) also using the standardized contact variables and the sample retrieved from the model including all variables as discussed above. However, as indicated earlier, the available sample for regressions including information from the SOEP 2014 wave is much smaller than the original IAB-SOEP Migration Sample, therefore, the sample sizes vary strongly between Model 1 and Model 2. To address these differences and increase comparability, I reran the former regressions with the sample used for the latter, following the same variable scheme (Model 3: Model 3.1, Model 3.2, Model 3.3). Model 3, therefore, includes the same sample as Model 2 which allows comparisons between the cross-sectional model and the lagged model.^4^

I used Wald-Chi-tests to compare the associations of national identification and the three standardized contact variables with each other. The Akaike Information Criterion (AIC) and Bayesian Information Criterion (BIC) were used to assess the overall fit of the models.

## Results

Of the 2,780 respondents from the IAB-SOEP subsample, 53% were male. The average age was almost 39 years, and the majority of respondents had completed secondary education (55%) and worked full-time (67%). With a mean of 12.2, the overall language skills were quite high. Overall, only 17% of respondents were second-generation immigrants, and almost half were German citizens (46%). Concerning origin, the largest group was composed by respondents originating from a member state of the European Union (39%) followed by respondents originating from member states of the Commonwealth of Independent States (28%). More detailed information on the sample can be found in Supplementary Appendix S4.

**FIGURE 1:**
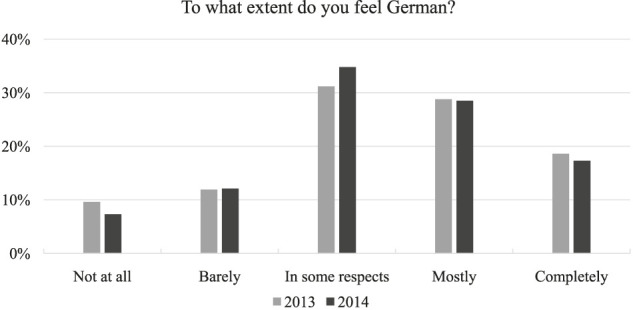
National identification in the years 2013 and 2014.

With regard to feeling German, 10% reported not feeling German at all, 12% reported feeling barely German, 31% felt German in some respects, 29% felt mostly German, and 19% felt completely German. As indicated by Figure 1, a similar distribution was found for the 2014 version of this variable. The most notable differences can be seen in the decreased percentages of respondents who indicated “not at all” and the increase in the “in some respects” category.

The three contact variables vary strongly in their distribution. While only 9% of respondents lived with a native family member, 28% reported that less than half of their friends are foreigners, and 51% of respondents reported that less than half of their co-workers are foreigners.

The starting point for the discussion of the link between national identification and the three contact variables is a multivariate cross-sectional analysis using all available cases. As discussed above, three models were run: a model without control variables, a model with general control variables, and a model including both general and migrant-specific control variables. The results, summarized in Table 1, indicate that the model including both general and migrant-specific control variables exhibited the best fit according to both the AIC and BIC. This was not only the case in the cross-sectional model with the larger sample, but across the three overall approaches (Model 1, Model 2, and Model 3). Therefore, in the following discussion of the results, I will focus on these models and only include information on the other models when noteworthy changes were observed following the inclusion of the control variables. Detailed information on all models can be retrieved from the respective tables.

**TABLE 1:** Cross-sectional ordered logit models.

	Model 1.1	Model 1.2	Model 1.3
Std native family	−0.05	−0.05	−0.05
	(−1.15)	(−1.23)	(−1.10)
Std mostly native friends	0.33***	0.34***	0.28***
	(8.43)	(8.45)	(6.79)
Std mostly native work	0.12**	0.15***	0.03
	(3.13)	(3.86)	(0.87)
Age	—	−0.01**	0.02***
	—	(−2.65)	(4.01)
Male	—	−0.25**	−0.11
	—	(−2.96)	(−1.23)
Secondary education	—	−0.08	−0.44*
	—	(−0.42)	(−2.36)
Higher education	—	−0.52**	−1.03***
	—	(−2.78)	(−5.30)
Marginal employment	—	−0.79***	−0.59***
	—	(−6.29)	(−4.69)
Part-time employment	—	−0.26*	−0.22*
	—	(−2.49)	(−2.03)
Language skills	—	—	0.18***
	—	—	(9.99)
Second generation	—	—	0.55***
	—	—	(4.92)
German citizenship	—	—	1.04***
	—	—	(11.76)
Turkey	—	—	−0.23*
	—	—	(−1.98)
CIS	—	—	0.52***
	—	—	(4.38)
Arab League	—	—	0.00
	—	—	(−0.02)
Other origin	—	—	0.43***
	—	—	(4.16)
Number of observations	2,402	2,402	2,402
Log likelihood	−3,578.93	−3,541.95	−3,292.33
AIC	7,171.87	7,109.89	6,624.67
BIC	7,212.36	7,185.08	6,740.35
Chi^2^ value: native family and mostly native friends	40.15***	41.56***	28.07***
Chi^2^ value: native family and mostly native work	7.94**	11.52***	1.86
Chi^2^ value: mostly native friends and mostly native work	13.00***	9.63**	15.83***

Notes: Std in the variable name indicates that the variable was standardized prior to the analyses, *z* statistics in parentheses, ^*^ *p* < 0.05, **p < 0.01, ***p < 0.001.

Regarding the link between contact and national identification, not all of the three contact variables had the expected positive and significant association. The link between having native family members and national identification was negative across all three models, however it was very small and statistically insignificant. This finding indicates that having native family members is, unlike expected, not associated with individual’s level of national identification. The association between having mostly native friends and national identification on the other hand was as expected, with respondents who have higher shares of native friends being more likely to report high levels of national identification. This relation was independent of the inclusion of control variables. The association with working in a predominantly native work setting, however, strongly depended on the inclusion of the migrant-specific control variables. When migrant-specific aspects were not controlled for, the association was positive, substantial, and statistically significant; upon the inclusion of these variables, the association was no longer significant and became quite small.

The comparison of the associations revealed that the association between national identification and having mostly native friends was significantly stronger than the relations between national identification and having native family members or large shares of native co-workers. The latter two associations, both statistically insignificant, did not differ in strength upon the inclusion of the migrant-specific control variables. Prior to the inclusion, having predominantly native co-workers appeared to be more strongly linked to national identification than having native family members.

As expected, most of the control variables—general and migrant-specific—had a statistically significant association with national identification. While the coefficient for age was positive and significant, it was quite small. The variables covering education and employment situation on the other hand all had strong negative associations with national identification. Concerning the migrant-specific variables, increased language skills, and being a second-generation migrant, as well as having the German citizenship were associated with higher levels of national identification. Out of the five regions of origin, respondents coded as originating from a CIS member state or a region belonging to the category “other origin” were more likely to report high levels of national identification than those originating from an EU member state.

The results obtained from Model 3, the models using the sample from the lagged analyses but the cross-sectional approach, were very close to the results from Model 1. The main associations, the directions, sizes of the coefficients, and significances only changed slightly between the models. Similarly, the conclusions about the overall fit were practically unchanged. Concerning the differences in the strength of the associations, the general results from Model 1 hold, with the exception that prior to the inclusion of migrant-specific control variables there was no statistically significant difference in the strength of the associations of having mostly native friends and having mostly native co-workers with national identification. Further, for some of the control variables noteworthy changes concerning the size and significance of the association with national identification were observed. While in the full model with the larger sample (Model 1.3) secondary education, part-time employment and being of Turkish origin were all negatively associated with national identification, this significant link could no longer be found in the smaller sample. Overall, the associations between national identification and the control variables appeared to be smaller in Model 3. However, because of the overall similarities, the results obtained from Model 3 will not be discussed further. Details on the models and results can be found in Appendix A2.

Like the change towards the smaller sample, changing the analyses strategy to the lagged variable approach (Model 2) yielded few changes in the results. In comparison to Model 3, the association between having native family members and national identification was positive, however it was still very small and stayed statistically insignificant (Model 2, Table 2). This was found independent of the inclusion of various control variables. Concerning the association between national identification and having a larger share of native friends, no substantial change was observed. The coefficients had the same size (in fact they were identical to the cross-sectional Model 1), meaning the relation was substantial. Respondents reporting lower shares of foreign friends in 2013 were significantly more likely to report high levels of national identification in 2014. Lastly, working in a predominantly native work setting once again had no significant association with national identification when migrant-specific control variables were included. Across the three models (Model 2.1, Model 2.2, and Model 2.3) the associations appeared to be very similar in size and significance to their equivalences in the cross-sectional models (Model 3.1, Model 3.2, and Model 3.3 in Appendix A2).

**TABLE 2:** Lagged ordered logit models.

	Model 2.1	Model 2.2	Model 2.3
Std native family	0.02	0.02	0.01
	(0.42)	(0.41)	(0.28)
Std mostly native friends	0.33***	0.34***	0.28***
	(7.01)	(7.14)	(5.76)
Std mostly native work	0.16***	0.19***	0.07
	(3.57)	(4.03)	(1.51)
Age	—	−0.01	0.02**
	—	(−1.59)	(3.13)
Male	—	−0.15	−0.02
	—	(−1.52)	(−0.17)
Secondary education	—	−0.10	−0.47
	—	(−0.44)	(−1.93)
Higher education	—	−0.50*	−1.01***
	—	(−2.13)	(−4.04)
Marginal employment	—	−0.87***	−0.51***
	—	(−5.88)	(−3.40)
Part-time employment	—	−0.07	0.05
	—	(−0.60)	(0.40)
Language skills	—	—	0.16***
	—	—	(7.37)
Second generation	—	—	0.31*
	—	—	(2.28)
German citizenship	—	—	1.23***
	—	—	(11.56)
Turkey	—	—	−0.27
	—	—	(−1.85)
CIS	—	—	0.14
	—	—	(1.03)
Arab League	—	—	0.21
	—	—	(0.98)
Other origin	—	—	0.39**
	—	—	(3.15)
Number of observations	1,675	1,675	1,675
Log likelihood	−2,437.80	−2,412.48	−2,258.29
AIC	4,889.59	4,850.96	4,556.58
BIC	4,927.56	4,921.47	4,665.05
Chi^2^ value: native family and mostly native friends	19.51***	20.49***	14.02***
Chi^2^ value: native family and mostly native work	4.31*	5.29*	0.68
Chi^2^ value: mostly native friends and mostly native work	5.65*	4.48*	8.18**

Notes: Std in the variable name indicates that the variable was standardized prior to the analyses, *z* statistics in parentheses, ^*^ *p* < 0.05, **p < 0.01, ***p < 0.001.

The comparison of the associations again drew a very clear picture in favor of the contact situation including native friends. The association between having predominately native friends and national identification was significantly larger than the respective associations regarding having native family members and having larger shares of native co-workers. The difference between the latter two was again insignificant upon the inclusion of the migrant-specific control variables. Prior to the inclusion, working in a predominantly native work setting appeared to be more strongly linked to national identification than having native family members.

The associations between the general and migrant-specific control variables and national identification mostly stayed the same upon applying the lagged approach. The only noteworthy change was that while coming from a CIS member state was significantly and substantially linked to national identification in the reduced cross-sectional model (Model 3.3.), this link could no longer be found in the lagged model (Model 2.3). For all other control variables, the significance structure stayed generally the same, with only small changes in the strength of the associations.

## Discussion

While previous research discussed the relevance of contact to natives for migrants’ national identification and tested this assumption in specific settings, little is known about the differences in effects of various kinds of contact. It is therefore unclear which kinds of contact are most strongly linked to national identification. Or assuming causality, which kinds of contact are most influential for migrants’ national identification. I gathered first indications concerning this relation by looking at the existing studies that have examined the link between national identification and various contact settings individually and by discussing the results of studies that included multiple contact settings as control variables.

Aspects of the concept of social distance in combination with the self-categorization theory, the social identity theory, and the contact theory suggested that, in general, migrants’ national identification should be positively associated with the amount of contact they have to natives (H1). However, extensions of the contact theory suggest that for attitudinal changes to occur, the contact situation needs to meet specific requirements (Amir, 1969; Cook, 1978; Brewer and Miller, 1984). I argued that these requirements were more likely to be met in certain contact situations. Specifically, I discussed three kinds of contacts: contact within the family setting, contact with friends, and contact within the workplace. For each of these settings, the fulfillment of the requirements was discussed. Overall, theoretical considerations suggested that the requirements for attitudinal change were most often met within the family setting followed by migrants’ contact with friends. In contrast, for work-related contacts, the number of requirements met was presumably lower. Therefore, I expected the association between having native family members and national identification to be greater than the association between having native friends (H2) or native coworkers (H3) and national identification. Similarly, having native friends was expected to be more strongly linked to national identification than having native coworkers (H4).

Contrary to expectations, the results indicated that the link between national identification and contact to natives was not generally positive. Rather, the existence of the association was highly dependent on the specific contact situation. Therefore, H1 could not be corroborated.

Concerning the link between having native family members and national identification, neither the cross-sectional models nor the lagged models’ results supported the generally expected positive association. Migrants with native family members were not more likely to report high levels of national identification than migrants without native family members. Therefore, it seems as if there is no link between having native family members and national identification. Unlike the family aspect, having mostly native friends was positively associated with national identification in all models, that is, cross-sectional and lagged. Further, this association was significantly larger than the insignificant association between having native family members and national identification. Therefore, H2 had to be rejected. H4, in contrast, could be corroborated since the association between having predominantly native coworkers and national identification was significantly smaller than the association between having predominantly native friends and national identification. However, it should be mentioned that the link between having a larger share of native co-workers and national identification was insignificant in both the full cross-sectional and the full lagged model. Upon closer inspection, it became clear that the link between the two variables depended highly on the inclusion of the control variable related to the respondents’ language skills. Lastly, H3 suggested that the association with having native family members would be larger than the association with working in a predominantly native work setting. Contrary to the expectations, the associations, both being insignificant, did not differ significantly from each other in the full model. In the models without the migrant-specific control variables the associations differed significantly, however, the association between working with larger shares of native co-workers and national identification consequently appeared to be larger.

Drawing from these results, it can be concluded that contact to native friends is the most relevant form of contact to natives with regard to migrants’ national identification. Programs aiming to increase national identification or, more generally, emotional integration among migrants should therefore focus on settings in which friendships between migrant and native participants can be formed.

This study, however, is not without limitations. One issue concerns the measure for national identification. The concept was measured by a single question: “To what degree do you think of yourself as German?”. No further indicators for national identification were available in the data set. Therefore, no indices covering multiple aspects of national identification or similar approaches could be applied and the possibilities to validate the instrument were severely restricted. Future research should aim towards creating a measure which includes multiple aspects of national identification and apply this measure in the replication of studies analyzing the relation between aspect of social integration and national identification.

Besides that, the variable measuring the contact to natives within the family setting should be discussed. The sample is very unbalanced regarding the family setting, less than 10% of the sample were coded as living with a native family member. Since the family setting variable was constructed from information on the migration status of migrants’ participating family members, it is possible that migrants who were coded as living without a native family member actually had native family members who simply chose not to participate in the study. An equal participation rate across non-migrant and migrant family members needs to be assumed for a valid interpretation of the results. However, there is no information backing the assumption.

Further, while lagged models were used, no full longitudinal analyses were possible since the contact and migrant-specific variables were only collected in the IAB-SOEP Migration Sample and not in later waves of the SOEP. This also meant that no analyses regarding the reversed causation was possible. It is therefore unclear whether the link between having native friends and national identification is solely based on the effect of contact on national identification or if the migrants’ level of national identification might also influence their choice of friends.

In conclusion, there are still a few issues that require further attention, such as the small sample of migrants with native family members and the topic of (reversed) causation. Nonetheless, the presented study provides valuable insights into the field: first, by presenting an overview of the existing literature on the influence of social integration on national identification; second, by offering a theoretical approach linking the two aspects; and finally, by simultaneously analyzing the associations between national identification and contact to natives across multiple settings. Overall, for migrants in Germany, the formation of national identification was strongly linked to their friendships with natives, but not so much to their contact to natives within the family or workplace setting.

## Data Availability

Publicly available datasets were analyzed in this study. This data can be found here: https://www.diw.de/en/diw_01.c.678568.en/research_data_center_soep.html.

## Footnotes

1 The issue of causation is a general problem in many studies analyzing the relation between contact to natives and national identification. Most research so far, especially with regard to adults, used cross-sectional data and can therefore only report on the general link between the two concepts. Cross-sectional analyses are unable to identify whether it is having native friends that influences national identification or national identification that influences having native friends. An article by Leszczensky et al. (2019) provides an overview over this issue by highlighting theoretical considerations concerning the link between the two concepts as well as discussing studies using different methodological approaches. The latter with a special focus on studies with school children rather than adult samples.

2 With 1,109 individuals, the unemployed respondents with a migration background represent a group of significant size. As a robustness check I ran additional models including these respondents and only the relations between national identification and the two social spheres for which information was available for this group (family and friends). Overall, the coefficients were similar in direction, size, and significance. Further, the comparison between the coefficients yielded the same results as the overall analyses below. More information can be found in Supplementary Appendix S1.

3 Different operationalizations were applied in robustness checks. Concerning the results, coding those who reported “about half” their friends/co-workers to be foreigners as 1, rather than 0, did not lead to substantial differences in the overall conclusion. Similarly, assuming an underlying interval scale and using the full information provided by the original measure led to coefficients comparable to those reported below. Further information on the robustness checks can be found in Supplementary Appendix S1.

4 Additionally, as a robustness check, I ran separate models for the three spheres. These models indicate the main relation between each kind of contact and national identification. Supplementary Appendix S3 shows the results for the three lagged models with control variables.

## Appendix

**TABLE A1:** Sample development.

Change	Lost	Left
Original sample		4,964
Dropped: no migration background	309	4,655
Dropped: unemployed	1,609	3,046
Dropped: apprentices	240	2,806
Dropped: students	22	2,784
Dropped: voluntary social year	3	2,781
Dropped: retirees working 0 hours	1	2,780
Sample for cross-sectional analyses		2,780
Merge with SOEP 2014	837	1,943
Sample for lagged analyses		1,943

**TABLE A2:** Cross-sectional ordered logit models with sample from lagged models.

	Model 3.1	Model 3.2	Model 3.3
Std native family	−0.06	−0.05	−0.08
	(−1.18)	(−1.14)	(−1.55)
Std mostly native friends	0.31***	0.31***	0.28***
	(6.51)	(6.54)	(5.63)
Std mostly native work	0.17***	0.20***	0.06
	(3.68)	(4.21)	(1.26)
Age		−0.01	0.02***
		(−1.78)	(3.63)
Male		−0.14	0.01
		(−1.39)	(0.06)
Secondary education		−0.02	−0.31
		(−0.07)	(−1.24)
Higher education		−0.46	−0.88***
		(−1.85)	(−3.43)
Marginal employment		−1.01***	−0.65***
		(−6.77)	(−4.35)
Part-time employment		−0.15	−0.08
		(−1.24)	(−0.67)
Language skills			0.18***
			(8.32)
Second generation			0.43**
			(3.17)
German citizenship			0.94***
			(8.94)
Turkey			−0.18
			(−1.22)
CIS			0.45**
			(3.28)
Arab league			−0.19
			(−0.86)
Other origin			0.51***
			(4.17)
Number of observations	1675	1675	1675
Log likelihood	−2484.93	−2452.50	−2305.57
AIC	4983.86	4931.00	4651.15
BIC	5021.82	5001.50	4759.62
Chi^2^ value: native family and mostly native friends	27.19***	27.27***	24.22***
Chi^2^ value: native family and mostly native work	10.77**	13.40***	3.73
Chi^2^ value: mostly native friends and mostly native work	3.93*	2.57	8.63**

Notes: Std in the variable name indicates that the variable was standardized prior to the analyses, z statistics in parentheses, *p < 0.05, **p < 0.01, ***p < 0.001.
