# Supplementary material for: Migrants’ Social Integration and Its Relevance for National Identification: An Empirical Comparison Across Three Social Spheres
Source: Front Sociol. 2022 Jan 3;6:700580. doi: 10.3389/fsoc.2021.700580 (PMC8762104; doi:10.3389/fsoc.2021.700580)
Supplement: Supplementary file 1 [file Table1.pdf]

Migrants' social integration and its relevance for national identification: An empirical comparison across three social spheres

Supplementary Appendix

TABLE S1 | Robustness checks using the full lagged ordered logit approach.

	Sample includes the unemployed	„about half" coded as 1 in mostly native friends and mostly native work	Full scale instead of dummies for mostly native friends and mostly native work
Std native family	0.00	0.01	0.00
	(-0.03)	(0.23)	(-0.07)
Std mostly native friends	0.27***	0.29***	0.41***
	(7.01)	(5.99)	(7.56)
Std mostly native work		0.11*	0.08
		(2.38)	(1.69)
Age	0.02***	0.02***	0.02**
	(5.43)	(3.38)	(3.22)
Male	0.03	-0.08	-0.06
	(0.34)	(-0.74)	(-0.58)
Secondary education	-0.16	-0.55*	-0.50*
	(-1.05)	(-2.23)	(-2.05)
Higher education	-0.68***	-1.04***	-1.06***
	(-4.25)	(-4.15)	(-4.21)
Unemployed	0.02		
	(0.19)		
Marginal employment	-0.35**	-0.48**	-0.50**
	(-2.66)	(-3.14)	(-3.29)
Part-time employment	0.11	0.07	0.04
	(0.92)	(0.56)	(0.31)
Language skills	0.18***	0.15***	0.15***
	(10.96)	(6.67)	(6.66)
Second generation	0.29**	0.39**	0.35**
	(2.82)	(2.86)	(2.60)
German citizenship	1.16***	1.27***	1.22***
	(14.11)	(11.99)	(11.54)
Turkey	-0.28*	-0.27	-0.19
	(-2.50)	(-1.90)	(-1.31)
CIS	0.18	0.14	0.22
	(1.65)	(0.98)	(1.55)
Arab League	-0.09	0.15	0.24

	(-0.55)	(0.74)	(1.16)
Other origin	0.26**	0.37**	0.39**
	(2.73)	(3.03)	(3.16)
Number of observations	2,775	1,675	1,675
Log likelihood	-3800.23	-2255.01	-2244.27
AIC	7640.46	4550.02	4528.54
BIC	7759.03	4658.49	4637.01
Chi2 value: native family and mostly native friends	20.63***	15.47***	29.48***
Chi2 value: native family and mostly native work		2.07	1.45
Chi2 value: mostly native friends and mostly native work		6.33*	17.31***

Notes: Std in the variable name indicates that the variable was standardized prior to the analyses, z statistics in parentheses, * $p < 0.05$, ** $p < 0.01$, *** $p < 0.001$

TABLE S2 | Details on the regions of origin.

Region (used in the analyses)	Countries in the dataset
Turkey	Turkey
Commonwealth of Independent States (CIS)	Armenia, Azerbaijan, Belarus, Kazakhstan, Kirgizstan, Moldova, Russia, Tadzhikistan, Turkmenistan, Ukraine, Uzbekistan
Arab League	Algeria, Bahrain, Comoros, Djibouti, Egypt, Iraq, Jordan, Kuwait, Lebanon, Libya, Morocco, Mauritania, Oman, Palestine, Qatar, Saudi Arabia, Somalia, Sudan, Syria, Tunisia, United Arab Emirates, Yemen
European Union (EU)	Austria, Belgium, Bulgaria, Croatia, Cyprus, Czech Republic, Denmark, Estonia, Finland, France, Greece, Great Britain, Hungary, Ireland, Italy, Latvia, Lithuania, Luxembourg, Malta, Netherlands, Poland, Portugal, Romania, Slovakia, Slovenia, Spain, Sweden
Others	All other countries of origin

TABLE S3 | Separate lagged ordered logit models for the three spheres.

	Family Model	Friends Model	Work Model
Std native family	0.03		
	(0.68)		
Std mostly native friends		0.29^{***}	
		(6.07)	
Std mostly native work			0.11^{*}
			(2.42)
Age	0.02^{**}	0.02^{**}	0.02^{**}
	(3.14)	(3.05)	(3.25)
Male	-0.02	-0.02	-0.01
	(-0.15)	(-0.21)	(-0.14)
Secondary education	-0.46	-0.46	-0.46
	(-1.87)	(-1.90)	(-1.90)
Higher education	-0.92^{***}	-0.98^{***}	-0.97^{***}
	(-3.70)	(-3.92)	(-3.89)
Marginal employment	-0.50^{***}	-0.51^{***}	-0.50^{***}
	(-3.35)	(-3.38)	(-3.35)
Part-time employment	0.07	0.04	0.09
	(0.59)	(0.33)	(0.72)
Language skills	0.18^{***}	0.16^{***}	0.17^{***}
	(8.24)	(7.66)	(7.94)
Second generation	0.30^{*}	0.30^{*}	0.30^{*}
	(2.23)	(2.28)	(2.23)
German citizenship	1.28^{***}	1.24^{***}	1.26^{***}
	(12.08)	(11.65)	(11.90)
Turkey	-0.40^{**}	-0.28	-0.40^{**}
	(-2.81)	(-1.95)	(-2.81)
CIS	0.01	0.14	0.00
	(0.08)	(1.04)	(-0.01)
Arab League	0.11	0.19	0.11
	(0.50)	(0.90)	(0.55)
Other origin	0.36^{**}	0.40^{**}	0.34^{**}
	(2.93)	(3.24)	(2.80)
Number of observations	1675	1675	1675
Log likelihood	-2277.90	-2259.50	-2275.19
AIC	4591.79	4555.00	4586.37
BIC	4689.42	4652.62	4684.00

Notes: Std in the variable name indicates that the variable was standardized prior to the analyses, z statistics in parentheses, * $p < 0.05$, ** $p < 0.01$, *** $p < 0.001$

TABLE S4 | Variables used in the analyses and descriptive statistics.

Variable	Operationalization	Min	Max	M	SD
**Dependent variables**					
National identification 2013	5 categories ranging from not at all to completely	1	5	3.35	1.19
National identification 2014	5 categories ranging from not at all to completely	1	5	3.36	1.12
**Independent variables**					
Native family	= 1 if living together with someone who has no migration background, 0 otherwise	0	1	0.09	0.28
Mostly native friends	= 1 if about one-quarter, less than one-quarter or none of their friends were foreigners, 0 otherwise	0	1	0.28	0.45
Mostly native work	= 1 if about one-quarter, less than one-quarter or none of the staff at their workplace were foreigners, 0 otherwise	0	1	0.51	0.5
**Socio-economic control variables**					
Age	In years	18	72	38.95	10.29
Male	= 1 if male; 0 if female	0	1	0.52	0.50
Secondary education	= 1 if highest degree comes from secondary education institution	0	1	0.55	0.50
Higher education	= 1 if educated beyond secondary education, 0 otherwise	0	1	0.41	0.49
Part-time employment	= 1 if working part-time, 0 otherwise	0	1	0.21	0.41
Marginal employment	= 1 if marginally employed, 0 otherwise	0	1	0.12	0.32
**Migrant-specific control variables**					
Language skills	Index of self-reported skills on writing, reading, and speaking	0	15	12.20	2.69
Second generation	= 1 if born in Germany, 0 otherwise	0	1	0.17	0.38
German citizenship	= 1 if German citizenship, 0 otherwise	0	1	0.46	0.50
Turkey	= 1 if country of origin is Turkey, 0 otherwise	0	1	0.13	0.34

CIS	= 1 if country of origin is member of the CIS, 0 otherwise	0	1	0.28	0.45
Arab League	= 1 if country of origin is member of the Arab League, 0 otherwise	0	1	0.03	0.17
Other origin	= 1 if country of origin is not Turkey, CIS or Arab League or European Union member, 0 otherwise	0	1	0.17	0.37
